# Reproductive and sexual sequelae of neuroendocrine tumour therapies: an under-recognised challenge

**DOI:** 10.1530/ERC-25-0500

**Published:** 2026-04-15

**Authors:** Kalyan Mansukhbhai Shekhda, Preet Mukesh Shah, Aiyappa Biddanda, Ashley Grossman, Martyn Caplin

**Affiliations:** ^1^ Neuroendocrine Tumour Unit, ENETS Centre of Excellence, Royal Free Hospital, London, UK; ^2^ Department of Diabetes and Endocrinology, London North West University Healthcare NHS Trust, London, UK; ^3^ Department of Diabetes and Endocrinology, University College London Hospital, London, UK; ^4^ Professor of Endocrinology, University of Oxford, Oxford, UK; ^5^ Professor of Medicine and Gastroenterology, University College London, London, UK

**Keywords:** neuroendocrine tumours, reproductive health, pregnancy, somatostatin analogues, PRRT, sexual function

## Abstract

Neuroendocrine tumours (NETs) are heterogeneous, biologically variable tumours arising from the diffuse neuroendocrine system. While more frequently seen in older patients, the incidence and prevalence of NETs in the younger population are increasing. In addition, because of their generally slow progression and favourable prognosis, coupled with widely available effective treatments, survival times even with metastatic tumours may often be prolonged. However, there is a paucity of data on the effect of treatment of NETs on men’s and women’s reproductive and sexual health. In this review, we have evaluated the effects of NET therapies, including somatostatin analogues (SSTAs), molecular targeted therapy (everolimus and sunitinib), peptide receptor radionuclide therapy (PRRT), and chemotherapy, on reproductive and sexual function in patients with NETs. There is a lack of evidence on the detrimental effects of SSTAs on human fertility, and indeed, many patients have conceived successfully after many years of treatment with SSTAs. Even patients who have received SSTAs during pregnancy have generally shown positive maternal and fetal outcomes. While the effects of PRRT and molecular targeted therapy on fertility are as yet poorly defined, chemotherapy has a proven negative impact on fertility; thus, family and pregnancy planning are strongly recommended before the initiation of chemotherapy. Finally, data on the effects of NET treatment on sexual function are very limited; however, neuroendocrine tumours can express oestrogen receptors/progesterone receptors (ER/PR) or testosterone receptors (TR); thus, checking tumour tissue for ER/PR/TR status prior to considering hormonal therapy for sexual dysfunction should be considered but warrants additional studies.

## Introduction

Neuroendocrine tumours (NETs) are heterogeneous, relatively rare tumours arising from the cells of the diffuse neuroendocrine system, with an incidence of around 7 cases per 100,000 population per year (1). They are generally slow-growing and have an overall relatively good prognosis, although they are not infrequently metastatic at diagnosis. They are characterised by a heterogeneity of the site of origin (gastrointestinal, pancreatic, bronchial, ovarian, etc.) and biological or functional variability (functional hormone secreting associated with a syndrome or non-functional with no associated hormone secretion or syndrome). Although classically rare in the younger population, the incidence and prevalence of NETs in this population have increased over the years (2). Furthermore, they are described in younger patients in the context of heritable tumour syndromes, usually inherited in an autosomal dominant manner, such as multiple endocrine neoplasia (MEN) syndromes, tuberous sclerosis (TS), neurofibromatosis 1 (NF-1), and von Hippel–Lindau syndrome (VHL) (3). In addition, the delay in the age of desired fertility characteristic of Western societies renders concern regarding the impact of these tumours more significant (4, 5). Somatostatin analogues (SSTAs), such as depot formulations of octreotide and lanreotide, are the mainstay of treatment in patients with advanced well-differentiated NETs, not only to stabilise tumour growth but also for symptom control. Additionally, short-acting octreotide is useful for symptom control in uncontrolled functioning NETs (6, 7, 8, 9). Apart from SSTAs, other treatment options, including molecular targeted therapy (sunitinib, everolimus, etc.), peptide receptor radionuclide therapy (PRRT), and chemotherapy, have been approved for the treatment of advanced, unresectable, well-differentiated, metastatic NETs (10). While the presence of NETs and their functional syndromes may negatively impact the patients’ health-related quality of life (HRQoL) (11), a recent systematic review suggested that not all systemic treatments for NETs have a negative impact on HRQoL (12). Indeed, the NETTER-1 trial on PRRT showed a statistical improvement in several domains of HRQoL (12). Nevertheless, there is little doubt that some therapeutic manoeuvres may be detrimental to HRQoL even when therapeutically efficacious.

While sexual and reproductive health is a major component of overall health and well-being, questions related to sexual and reproductive health are not routinely a part of HRQoL in patients with NETs (12, 13, 14). Only few studies have explored sexual function and dysfunction in patients with these tumours, with varying results. Despite many studies demonstrating the importance of the preservation of fertility and reproductive health in other areas of cancer care, little is known regarding the effects of the treatment in patients with NETs. In this article, we have summarised information that is currently available, evaluating the effect of NET treatment on women’s and men’s reproductive health and sexual function.

### Summary of key points

- Neuroendocrine tumours are increasingly more prevalent in the young reproductive age population.
- Evidence for the effects of neuroendocrine tumour treatment on reproductive and sexual health is very limited.
- The use of somatostatin receptor analogues during pregnancy appears to be safe for mother and fetus.
- Young patients in the reproductive age group should be counselled for fertility and pregnancy planning prior to initiation of peptide receptor radionuclide therapy, molecular targeted therapy, and chemotherapy. Baseline hormonal and radiological evaluation, such as anti-Müllerian hormone (AMH), follicle-stimulating hormone (FSH), luteinising hormone (LH), antral follicle count (AFC) in women, and semen analysis in men, is advisable prior to starting systemic treatment.
- Fertility preservation strategies in women include oocyte cryopreservation and ovarian tissue cryopreservation. If these are not possible, then adjunctive measures, such as ovarian suppression with GnRH agonists, can be considered to limit ovarian toxicity due to chemotherapy. Fertility preservation strategies in men include sperm cryopreservation, testicular sperm extraction (TESE) and cryopreservation, and testicular tissue cryopreservation. Embryo cryopreservation can be an option for couples who would not accept the above options.
- Counselling about the potential impact of NET therapy on sexual health and dysfunction is often overlooked during clinical consultations. These discussions should be part of clinical consultations. Neuroendocrine tumours can also express ER/PR or testosterone receptors (TR); thus, checking tumour tissue for ER/PR/TR status prior to considering hormonal therapy for sexual dysfunction should be considered.

### Review criteria

This narrative review was based on the assessment of all available prospective studies, retrospective studies, review articles, case series, and case reports published up to July 2025 in PubMed, Embase, and CINAHL. The search was conducted with the following key words and/or the combinations: “fertility”, “male fertility”, “female fertility”, “gonadal function”, “male hypogonadism”, “sexual health”, “sexual dysfunction” and “sexual function”, “psychological factors” AND “neuroendocrine tumours”, “neuroendocrine neoplasms”, “NETs”, “carcinoid”, “neuroendocrine tumour treatment”, “chemotherapy”, “molecular targeted therapy”, “sunitinib”, “peptide receptor radionuclide therapy”, “PRRT” and “Lu-DOTA-Octreotide”.

### Effects of NET treatment on reproductive health

#### Somatostatin analogues (SSTAs)

Somatostatin (SST) is a polypeptide hormone that has anti-secretory, anti-proliferative, and anti-angiogenic properties (15). SST binds to five different types of G protein-coupled transmembrane somatostatin receptors (SSTRs) and has a short half-life of 1–3 min (16). Apart from normal human tissues, such as the gastrointestinal tract, pancreas, the genitourinary tract, lungs and brain, SSTRs are also expressed in tumour cells, especially in neuroendocrine tumour cells. SSTAs bind to SSTR-2a and SSTR-5 with strong and mild-to-moderate affinity, respectively. They have a longer half-life of approximately 90–120 min for immediate release formulations and up to 5 days for sustained release formulations (17). SSTAs modulate their effects by two distinct mechanisms: first, due to direct tumoural binding to SSTRs, and second, due to inhibition of growth factors and modulation of immune responses (15). Their suppressive effects on growth hormone and IGF-1 are established, but the effects on reproductive hormones (FSH/LH) remain controversial (18). There are very limited data on the possible effect of SSTs on reproductive organs and fertility. In 2001, Goddard and colleagues found that somatostatin showed anti-proliferative effects on porcine Sertoli cells (19). In 2013, Riaz and colleagues found expression of SSTRs 1–5 in mouse Sertoli cells, and treatment with somatostatin peptide resulted in apoptosis and cell cycle arrest (20). Aside from these animal studies, a few human studies have also demonstrated the presence of SSTRs in reproductive organs in men and a possible role for SSAs and SSTRs on germ cell development. In 2012, Unger *et al.* demonstrated the presence of SSTRs 1–5 in ovaries and SSTR-2a and SSTR-5 in human testes (21). Recently, Filizoglu *et al.* have analysed ^68^Ga-DOTATATE whole-body PET/CT scans in 93 disease-free paediatric patients. They found significant ^68^Ga-DOTATATE uptake in the parotid glands, thyroid gland, thymus, liver, adrenal glands, stomach, intestine, uterus, prostate, and testes (22). In terms of fertility in women, it is known that the growth hormone axis is important for the initiation of puberty and the promotion of follicular maturation, and the use of SSTAs might hamper the onset of fertility (23). However, it could be argued that, as most of the reported cases of the use of SSTAs during pregnancy patients have been on SSTAs prior to pregnancy, it is unlikely that SSTAs have major effects on fertility *per se* (23, 24). Data on the safety of SSTAs in the mother and the fetus during pregnancy are very limited, and most of the reports relate to patients treated for acromegaly. However, a recent case series published by Ratnayake and colleagues described a total of 22 pregnancies in 18 patients with neuroendocrine tumours. In 5 pregnancies, 4 patients had received SSTAs prior to pregnancy; in 4 pregnancies, they had continued SSTAs throughout pregnancy, while one patient opted not to take SSTAs during the first trimester of pregnancy. All pregnancy outcomes were uneventful, including normal fetal growth. One patient with an insulinoma had received short-acting octreotide for the treatment of hypoglycaemia (2). A recently published case report of a 41-year-old woman with a diagnosis of metastatic bronchial NET who had received octreotide LAR for 6 years prior to pregnancy stopped this during the first two trimesters and then restarted from 28 weeks of pregnancy: pregnancy outcomes for the mother and baby were uneventful (25). Additionally, as SSTAs are now the first-line treatment for well-differentiated low-grade NETs over molecular targeted therapy and chemotherapy, it can also be argued that infertility risk following NET therapy in these well-differentiated low-grade NETs is less compared to those who have high-grade NETs or neuroendocrine carcinomas who would need treatment with molecular targeted therapy or chemotherapy with a high risk of fertility impairment. Given the limited safety data during pregnancy, the FDA has included lanreotide and octreotide in class C (adverse effects detected on animal studies but inadequate studies in humans) and class B (no adverse effects detected on animal studies but inadequate studies in humans) categories, respectively. Although the Endocrine Society clinical guidelines suggest switching from long-acting to short-acting octreotide preparations prior to conception and stopping these during pregnancy in acromegaly, no specific guidelines are available for NETs in pregnancy (26). Therefore, the benefits and risks should be carefully assessed and discussed with the patient prior to any change in medications for NET patients, and decisions should be individualised. However, the present situation appears to be relatively sanguine regarding the use of SSTAs during pregnancy, where this appears to be therapeutically appropriate. In our clinical practice in our NET unit, an ‘ENETS Centre of Excellence’, we usually discontinue SSTAs in the first trimester, with a view to restarting them from the second trimester onwards in patients with non-functioning NETs, whereas in patients with functional NETs, we continue SSTAs throughout pregnancy as the benefits of continuing them outweigh the small theoretical risk of materno-fetal complications during pregnancy. Additionally, around 30% of NETs, especially gastrointestinal NETs and breast NETs, have immunohistochemical (IHC) evidence of oestrogen receptors (ER) and/or progesterone receptors (PR) (27). As pregnancy is a high oestrogen/progesterone state, it may be useful to assess the presence of oestrogen and progesterone receptor status by immunocytochemistry, although a recent study on NET primary cell cultures did not reveal any consistent effect of oestrogens (28). Although excretion of SSTAs in breast milk has not been formally studied in humans, as a result of their pharmacological properties, such as being a high molecular weight peptide and being easily digestible in the stomach, it appears to be safe to use in lactating mothers. If the dose and frequency of SSTAs are frequent, careful monitoring of the infant is advisable, especially within the first six months (29).

#### Peptide receptor radionuclide therapy (PRRT)

This modality utilises the concept of targeting somatostatin receptors on the target tumour cells with specific radionuclides: ^90^Y-tetra-azacyclododecanetetra-acetic acid-D-Phe1-Tyr3-octreotide (^90^Y-DOTATOC) and ^177^Lu-tetra-azacyclododecanetetra-acetic acid-tyrosine3-octreotate (^177^Lu-DOTATATE) are the two most commonly used agents in this regard. Both of these are used for midgut NETs, whereas the latter agent is used in treating pancreatic and pulmonary NETs. In the case of gastrointestinal NETs, the latter is preferred due to its reduced haemotoxicity and nephrotoxicity characteristics (30, 31). For pulmonary NETs, the data on the use of ^90^Y-DOTATOC are limited, albeit mostly favourable, with a higher incidence of nephrotoxicity compared to ^177^Lu-DOTATATE (32). The gonads, being radiosensitive and due to their expression of SSTRs, could be adversely affected by these agents. Teunissen *et al.* observed transient fluctuations in FSH and inhibin B in men, but they also reported a sustained reduction in testosterone levels and sex hormone-binding globulin (SHBG) with a reciprocal rise in FSH levels, following treatment with ^177^Lu-DOTATATE (33). Zhang *et al.* documented that some women were able to conceive naturally despite receiving PRRT (34). The shortest interval between the last dose of PRRT and conception was 12 months, with the longest being 84 months (34). In their series, Ratnayake and colleagues reported one patient who conceived within a year of receiving PRRT (2). The case of a 41-year-old woman previously discussed had also received PRRT some four years prior to pregnancy (25). In 2024, the FDA approved PRRT treatment for adolescent patients 12 years of age and older, which implies that a better understanding of the effects of PRRT on future fertility in such younger patients is essential. A recent observational cohort study involved patients younger than 18 years with metastatic neuroendocrine cancers who received ^131^I-MIBG and/or ^177^Lu-DOTATATE; however, the effects of these therapies on the reproductive hormones were not studied (35). Regarding fertility, ^177^Lu-DOTATATE can have negative effects, in a dose-dependent manner, which can lead to temporary or permanent infertility (36). Pregnancy is considered an absolute contraindication, and breastfeeding can be considered a relative contraindication in women receiving PRRT (37). Pregnancy status must clearly be determined in women in the reproductive age group who are planned to receive PRRT (37). Effective birth control is recommended for women receiving PRRT and for seven months thereafter. Men with partners who are able to conceive are urged to use contraception for at least four months following the completion of therapy (36, 38). Although anti-Müllerian hormone (AMH) levels are advisable only for women prior to undergoing gonadotoxic chemotherapy for cancers (39), baseline investigations for ovarian reserve, such as anti-Müllerian hormone (AMH), follicle-stimulating hormone (FSH), luteinising hormone (LH), and antral follicle count (AFC), could be valuable in young women prior to initiation of PRRT; the other options for fertility preservation, such as cryopreservation of ovum and sperm, and later *in vitro* fertilisation (IVF), should be discussed prior to treatment with PRRT.

#### Chemotherapy

Many chemotherapy agents can not only cause iatrogenic premature ovarian insufficiency in women but also hamper fertility in both men and women (40). The chemotherapy agents most commonly used in the management of NETs and neuroendocrine carcinomas (NECs) include temozolomide, fluorouracil, capecitabine, streptozotocin, platinum-based agents (cisplatin, carboplatin, and oxaliplatin), etoposide, and irinotecan.

Temozolomide impairs spermatogenesis and disrupts oocyte viability, potentially inducing epigenetic changes that culminate in infertility (40, 41). Fluorouracil’s effects are debatable, with some studies linking it to ovarian developmental arrest, ovarian tissue damage, hormonal imbalances, and reduced sperm counts, while others have reported a minimal impact on fertility (42, 43). Capecitabine shows limited evidence of serious gonadal harm (43). Platinum-based compounds consistently trigger follicular apoptosis, deplete ovarian reserve, and cause azoospermia (44). Etoposide, especially in combination regimens, has been associated in animal models with fetal ovarian toxicity, oligospermia/azoospermia in men, and variable ovarian dysfunction in women (45, 46, 47). Irinotecan, in rodent studies, induces ovarian cell death, vascular compromise, long-term declines in ovarian weight and follicle numbers, as well as testicular cell death, reduced sperm production, decreased testicular mass, and elevated AMH levels in men (48). All these agents are, therefore, to be regarded as likely to cause severe gonadal damage, and, where appropriate, referral to reproductive specialists and fertility preservation techniques (sperm and ovarian preservation and IVF) should be considered and discussed (49).

#### Molecular targeted therapy

The targeted therapies that have been approved for use in NETs include everolimus (an mTOR inhibitor), sunitinib (a tyrosine kinase inhibitor), and, the most recent addition, cabozantinib (a tyrosine kinase inhibitor). Everolimus, used in gastro-pancreatic, lung, and unknown origin NETs, exerts a primary hypogonadotrophic effect and can cause erectile dysfunction in men but with insufficient data on women’s fertility (50). Sunitinib, indicated for pancreatic NETs, has been shown in experimental studies to inhibit spermatogenesis and suppress the hypothalamic–pituitary–adrenal axis in men, as well as to block ovulation in women (51, 52). There are no human fertility data on cabozantinib, employed for both pancreatic and extra-pancreatic NETs, but it induces oligospermia and ovarian necrosis in rat models (53). However, there are no specific data related to the effects of molecular targeted therapy on fertility and reproductive health when used in NET patients. There are insufficient data on the effect of everolimus when used in other cancers. Women of childbearing potential are advised to use highly effective contraception during everolimus therapy and for at least eight weeks after the last dose. Men should employ barrier methods during treatment and for at least four weeks post-treatment to prevent drug-related teratogenic risk via seminal fluid concentrations (https://www.ema.europa.eu/en/medicines/human/EPAR/votubia). In a small study involving the use of sunitinib in men with metastatic renal cell carcinoma (mRCC), secondary hypogonadism was seen (54). Women of childbearing potential are advised to use effective contraception during treatment with sunitinib and for ≥4 weeks after the last dose, and men must employ barrier methods during treatment and for ≥7 weeks post-treatment to mitigate any possible teratogenic risk (https://www.pfizermedical.com/sutent/population-use). There are insufficient data on the effect of cabozantinib when used in other cancers. Women of childbearing potential and female partners of men should use reliable contraception during treatment and for at least four months after the last dose; specifically, cabozantinib may reduce hormonal‐contraceptive efficacy, so barrier or non-hormonal methods should be used (http://www.bccancer.bc.ca/drug-database-site/Drug%20Index/Cabozantinib_monograph.pdf).

**Figure 1:**
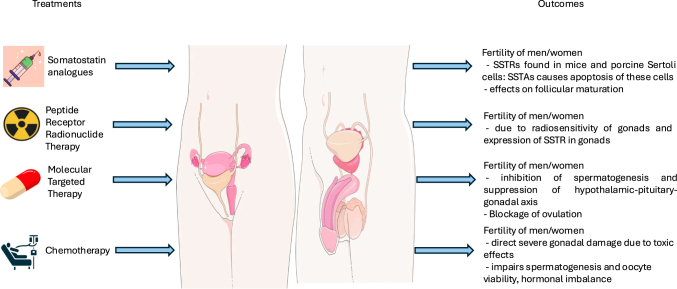
Effects of neuroendocrine tumour treatment on fertility in men and women.

**Figure 2:**
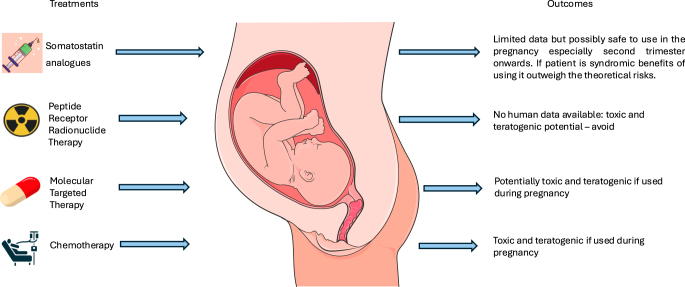
Effects of neuroendocrine tumour treatment on pregnancy outcomes.

Figures 1 and 2 describe the effects of neuroendocrine tumour therapy on reproductive health.

### Neuroendocrine tumour treatment and sexual health

Sexual dysfunction refers to the lack of sexual desire and function and to changes in the psychophysiological responses in patients. Some form of sexual dysfunction has a high prevalence in the general population, but not enough data are available on the prevalence of sexual dysfunction in cancer patients generally (55). In general, post-chemotherapy patients often show prolonged fatigue, cognitive limitations, chronic pain, insomnia, and anxiety, all of which play a significant role in inducing sexual dysfunction. These symptoms may persist for years after cancer treatment, and both general practitioners and endocrinologists should actively seek out these symptoms and offer appropriate care and counselling (55, 56, 57, 58). NETs and their treatments can make a significant contribution to both men’s and women’s sexual dysfunction; however, only a few studies have explored sexual health specifically in patients with NETs. Reassuringly, in the few published studies, it was observed that there was no difference in the prevalence of sexual dysfunction in NET patients compared to controls, nor was there any effect of NET treatment on sexual function. The authors had used the questionnaire for screening sexual dysfunction (QSD) score and the Patient-Reported Outcomes Measurement Information System (PROMIS). However, the sample sizes in these studies were small (59, 60). Karppinen and colleagues studied the HRQoL in 134 patients with gastrointestinal NETs who had advanced metastatic disease and who had received various treatments: they used the 15D questionnaire, but only a single question related to sexual activity was included. Overall, HRQoL was significantly impaired in patients compared to controls. Patients scored worse on 9 of 15 dimensions, which included sexual dysfunction. The dimension of sexual dysfunction was statistically significant in patients compared to controls (61). Apart from these, there are a few case reports mentioning symptoms related to sexual dysfunction (62, 63). A recent systematic review done by Gosain *et al.* on the effect of cancer therapeutics, in general, on HRQoL noted that most of the studies used commonly used questionnaires (e.g. EORTC QLQ-C30, EORTC QLQ-GI.NET21, and FACT-G), which lacked questions related to sexual health and libido (12). Generally, sexual dysfunction in men can only be accurately determined after administering a validated questionnaire, such as IIEF-6 (International Index of Erectile Function-6) or SHIM (Sexual Health Inventory for Men), and it is crucial to understand the basis on which each of these patient-reported outcome measures has been developed (64). Valid assessment of sexual dysfunction in women is even more complex. DSM-11 criteria now classify sexual dysfunction independent of gender description. The first step is to explore whether there are underlying endocrine disturbances that might be contributing to the dysfunction, which should then be followed by administering the relevant questionnaires to better understand the psycho-social factors involved in the dysfunction. Any confirmed endocrine disturbance should be appropriately treated. Women may benefit from changing their contraceptive pills to see if there might be a resurgence of libido. Hormone therapy as such is generally not recommended in women in the absence of a clear defect, but this can certainly be considered when the patient is menopausal (65). Men will benefit from an endocrine assessment with a further cardiovascular risk assessment. They can trial PDE5 inhibitors along with referral to urology services (66). However, in the first instance, it is essential to assess baseline hormonal parameters and consider treating any disorders, as for other patients with endocrine dysfunction. It should also be noted that as neuroendocrine tumour could express ER/PR or testosterone receptors (TR), checking tumour tissue for ER/PR/TR status prior to considering hormonal therapy for sexual dysfunction would aid in determining the theoretical risk of hormonal therapy.

**Figure 3:**
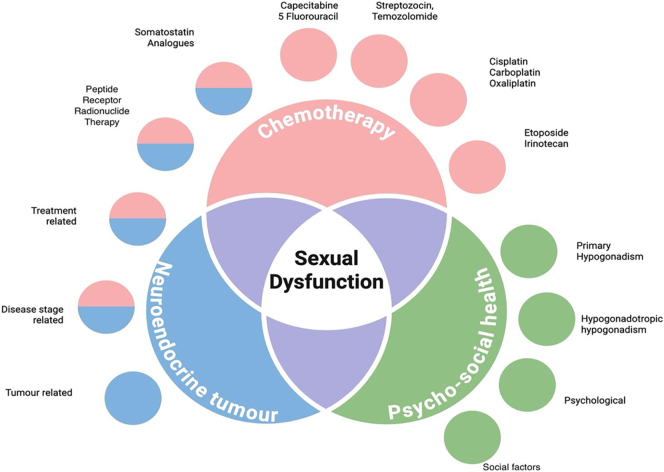
Complex interplay of neuroendocrine tumours, their treatment, and psychosocial health on sexual dysfunction in patients with NETs.

Figure 3 depicts how sexual dysfunction in patients with NETs develops due to the complex interplay of cancer, psycho-social factors, and effects of NET treatment.

## Conclusion

There is little known regarding the effects of NET treatment on fertility and sexual health. Despite animal studies suggesting that SSTAs might affect fertility, there is little clinical evidence to support this. There is growing evidence of mainly case reports and expert experience suggesting the safety of SSTAs during pregnancy; however, the small theoretic possibility of teratogenicity exists. Each patient should have an open discussion as to the possible risks of continuing the SSTAs, especially during the first trimester of pregnancy, considering whether SSTAs are required for syndromic hormonal control. Regarding PRRT, its use is contraindicated during pregnancy or while conception is being considered, and any attempt to conceive must be delayed for preferably at least 6 months after the completion of treatment. In addition, the use of PRRT in adolescents and possibly children should be tempered by the fact that we have little or no knowledge of its impact on future fertility. Chemotherapy effects on fertility are well established for other cancers, and the possibility of cryopreservation and possibly ovarian preservation should be seriously discussed upfront in men and women of reproductive age. There are little or no data on the effects of molecular targeted agents on fertility, but their use should generally be avoided unless there is effective contraception. Additional studies are needed. It is recommended that patients in the reproductive age group should have discussions about fertility, sexual health, family, and pregnancy planning prior to initiating these treatments. Checking tumour tissue for ER/PR/TR status prior to considering hormonal therapy for sexual dysfunction should also be considered. Many QoL questionnaires lack questions related to sexual health, and this needs addressing as well as further prospective studies to assess the effects of treatment of NETs on sexual health.

## Declaration of interest

MC received research funding from Ipsen and Novartis and serves on advisory boards for Ipsen, Novartis, IMT Pharma, and Crinetics. The other authors do not have any financial or potential conflicts of interest.

## Funding

This research did not receive any specific grant or funding from any funding agency in the public, commercial, or not-for-profit sector.

## Author contribution statement

All authors have contributed equally for this review article.

## References

[1] Dasari A, Shen C, Halperin D, et al. Trends in the incidence, prevalence, and survival outcomes in patients with neuroendocrine tumors in the United States. JAMA Oncol 2017 3 1335. (10.1001/jamaoncol.2017.0589) 28448665 PMC5824320

[2] Ratnayake GM, Shekhda KM, Glover T, et al. Neuroendocrine tumours and pregnancy: real‐world data from an European neuroendocrine Tumour *Centre of Excellence*. J Neuroendocrinol 2025 37 e13465. (10.1111/jne.13465) 39503166 PMC11750320

[3] Gaal J & de Krijger RR. Neuroendocrine tumors and tumor syndromes in childhood. Pediatr Dev Pathol 2010 13 427–441. (10.2350/09-04-0635-CP.1) 19708762

[4] Martin JA, Hamilton BE & Osterman MJK. Births in the United States, 2023. In: NCHS Data Brief, no 507. Hyattsville, MD: National Center for Health Statistics. 2024. (10.15620/cdc/158789) PMC11615961 39325585

[5] Mathews TJ & Hamilton BE. Mean age of mothers is on the rise: United States, 2000–2014. NCHS Data Brief 2016 232 1–8. 26828319

[6] Caplin ME, Pavel M, Ćwikła JB, et al. Lanreotide in metastatic enteropancreatic neuroendocrine tumors. N Engl J Med 2014 371 224–233. (10.1056/NEJMoa1316158) 25014687

[7] Rinke A, Wittenberg M, Schade-Brittinger C, et al. Placebo-controlled, double-blind, prospective, randomized study on the effect of octreotide LAR in the control of tumor growth in patients with metastatic neuroendocrine midgut tumors (PROMID): results of long-term survival. Neuroendocrinology 2017 104 26–32. (10.1159/000443612) 26731483

[8] Adams RL, Adams IP, Lindow SW, et al. Inhibition of endothelial proliferation by the somatostatin analogue SOM230. Clin Endocrinol 2004 61 431–436. (10.1111/j.1365-2265.2004.02098.x) 15473874

[9] Shekhda KM, Armeni E, Mandair D, et al. Octreotide infusion pump in patients with functional neuroendocrine tumors and refractory hormonal syndrome. Endocr Oncol 2025 5 e250016. (10.1530/EO-25-0016) 40384778 PMC12084797

[10] Pavel M, O’’Toole D, Costa F, et al. ENETS consensus guidelines update for the management of distant metastatic disease of intestinal, pancreatic, bronchial neuroendocrine neoplasms (NEN) and NEN of unknown primary site. Neuroendocrinology 2016 103 172–185. (10.1159/000443167) 26731013

[11] Scandurra C, Modica R, Maldonato NM, et al. Quality of life in patients with neuroendocrine neoplasms: the role of severity, clinical heterogeneity, and resilience. J Clin Endocrinol Metab 2021 106 e316–e327. (10.1210/clinem/dgaa760) 33084861

[12] Gosain R, Gupta M, Roy AM, et al. Health-related quality of life (HRQoL) in neuroendocrine tumors: a systematic review. Cancers 2022 14 1428. (10.3390/cancers14061428) 35326587 PMC8946839

[13] Vasconcelos P, Carrito ML, Quinta-Gomes AL, et al. Associations between sexual health and well-being: a systematic review. Bull World Health Organ 2024 102 873–887D. (10.2471/BLT.24.291565) 39611198 PMC11601183

[14] Fayers P & Bottomley A. Quality of life research within the EORTC – the EORTC QLQ-C30. Eur J Cancer 2002 38 125–133. (10.1016/S0959-8049(01)00448-8) 11858978

[15] Rai U, Thrimawithana TR, Valery C, et al. Therapeutic uses of somatostatin and its analogues: current view and potential applications. Pharmacol Ther 2015 152 98–110. (10.1016/j.pharmthera.2015.05.007) 25956467

[16] O’Toole TJ & Sharma S. Physiology, Somatostatin. In StatPearls [Internet]. Treasure Island, FL, USA: StatPearls Publishing, 2025. (https://www.ncbi.nlm.nih.gov/books/NBK538327/) 30855911

[17] Anthony L & Freda PU. From somatostatin to octreotide LAR: evolution of a somatostatin analogue. Curr Med Res Opin 2009 25 2989–2999. (10.1185/03007990903328959) 19842996 PMC3678951

[18] Csaba Z & Dournaud P. Cellular biology of somatostatin receptors. Neuropeptides 2001 35 1–23. (10.1054/npep.2001.0848) 11346306

[19] Goddard I, Bauer S, Gougeon A, et al. Somatostatin inhibits stem cell factor messenger RNA expression by Sertoli cells and stem cell factor-induced DNA synthesis in isolated seminiferous tubules. Biol Reprod 2001 65 1732–1742. (10.1095/biolreprod65.6.1732) 11717135

[20] Riaz H, Liang A, Khan MK, et al. Somatostatin and its receptors: functional regulation in the development of mice Sertoli cells. J Steroid Biochem Mol Biol 2013 138 257–266. (10.1016/j.jsbmb.2013.06.007) 23831358

[21] Unger N, Ueberberg B, Schulz S, et al. Differential expression of somatostatin receptor subtype 1–5 proteins in numerous human normal tissues. Exp Clin Endocrinol Diabetes 2012 120 482–489. (10.1055/s-0032-1314859) 22976314

[22] Filizoglu N, Ozguven S, Kesim S, et al. Physiological bio-distribution of 68Ga-DOTA-TATE in pediatric patients. Ann Nucl Med 2025 39 650–662. (10.1007/s12149-025-02040-9) 40106206 PMC12174203

[23] Zamponi V, La Salvia A, Tarsitano MG, et al. Effect of neuroendocrine neoplasm treatment on human reproductive health and sexual function. J Clin Med 2022 11 3983. (10.3390/jcm11143983) 35887747 PMC9324753

[24] Pistilli B, Grana C, Fazio N, et al. Pregnant with metastatic neuroendocrine tumour of the ovary: what now? Ecancermedicalscience 2012 6 240. (10.3332/ecancer.2012.240) 22331988 PMC3273852

[25] Constantin AE, Cirstoiu MM, Draghici G, et al. A multidisciplinary approach to managing carcinoid syndrome in pregnant women: ensuring maternal and fetal safety trough interdisciplinary collaboration. Medicina Moderna Mod Med 2025 32 193–202. (10.31689/rmm.2025.32.2.193)

[26] Katznelson L, Laws ER, Melmed S, et al. Acromegaly: an endocrine society clinical practice guideline. J Clin Endocrinol Metab 2014 99 3933–3951. (10.1210/jc.2014-2700) 25356808

[27] Barros MJ, Strosberg J, Al-Toubah T, et al. HORMONET: a phase II trial of tamoxifen for estrogen/progesterone receptor-positive neuroendocrine tumors. Ther Adv Med Oncol 2023 15 17588359231186040. (10.1177/17588359231186041) PMC10387775 37529158

[28] Wang K, Fischer A, Maccio U, et al. Impact of sex hormones on pheochromocytomas, paragangliomas, and gastroenteropancreatic neuroendocrine tumors. Eur J Endocrinol 2025 192 46–60. (10.1093/ejendo/lvae163) 39804847

[29] Drugs and Lactation Database (LactMed®) [Internet]. Octreotide. Bethesda, MD: National Institute of Child Health and Human Development, 2006. [Updated 2025 Mar 15]. (https://www.ncbi.nlm.nih.gov/books/NBK501724/)

[30] Strosberg J, El-Haddad G, Wolin E, et al. Phase 3 trial of ^177^ Lu-Dotatate for midgut neuroendocrine tumors. N Engl J Med 2017 376 125–135. (10.1056/NEJMoa1607427) 28076709 PMC5895095

[31] Yalchin M, Oliveira A, Theocharidou E, et al. The impact of radiological response to peptide receptor radionuclide therapy on overall survival in patients with metastatic midgut neuroendocrine tumors. Clin Nucl Med 2017 42 e135–e141. (10.1097/RLU.0000000000001457) 27922860

[32] Mariniello A, Bodei L, Tinelli C, et al. Long-term results of PRRT in advanced bronchopulmonary carcinoid. Eur J Nucl Med Mol Imaging 2016 43 441–452. (10.1007/s00259-015-3190-7) 26392198

[33] Teunissen JJM, Krenning EP, de Jong FH, et al. Effects of therapy with [177Lu-DOTA0,Tyr3]octreotate on endocrine function. Eur J Nucl Med Mol Imaging 2009 36 1758–1766. (10.1007/s00259-009-1151-8) 19471926 PMC2764054

[34] Zhang J, Kulkarni HR, Lehmann C, et al. Pregnancy and delivery after PRRT without sequelae. Clin Nucl Med 2018 43 842–845. (10.1097/RLU.0000000000002267) 30179916

[35] Peet C, Elmaraghi C, Abdel-Aziz T, et al. Molecular radiotherapy for adult type metastatic neuroendocrine tumours in children. Eur J Nucl Med Mol Imaging 2025 24 4016–4024. (10.1007/s00259-025-07247-6) PMC12397108 40272497

[36] Shaheen S, Moradi F, Gamino G, et al. Patient selection and toxicities of PRRT for metastatic neuroendocrine tumors and research opportunities. Curr Treat Options Oncol 2020 21 25. (10.1007/s11864-020-0711-9) 32172368

[37] Bodei L, Cremonesi M, Grana CM, et al. Peptide receptor radionuclide therapy with 177Lu-DOTATATE: the IEO phase I–II study. Eur J Nucl Med Mol Imaging 2011 38 2125–2135. (10.1007/s00259-011-1902-1) 21892623

[38] Kendi AT, Halfdanarson TR, Packard A, et al. Therapy with ^177^ Lu-DOTATATE: clinical implementation and impact on care of patients with neuroendocrine tumors. Am J Roentgenol 2019 213 309–317. (10.2214/AJR.19.21123) 31039017

[39] Gordon CE & Yanushpolsky E. Anti-Müllerian hormone: current understanding and clinical use. Curr Obstet Gynecol Rep 2021 10 61–70. (10.1007/s13669-021-00310-7)

[40] Stone JB, Kelvin JF & DeAngelis LM. Fertility preservation in primary brain tumor patients. Neuro Oncol Pract 2017 4 40–45. (10.1093/nop/npw005) PMC5815629 29479452

[41] Berthaut I, Montjean D, Dessolle L, et al. Effect of temozolomide on male gametes: an epigenetic risk to the offspring? J Assist Reprod Genet 2013 30 827–833. (10.1007/s10815-013-9999-8) 23652788 PMC3696451

[42] Naren G, Guo J, Bai Q, et al. Reproductive and developmental toxicities of 5-fluorouracil in model organisms and humans. Expert Rev Mol Med 2022 24 e9. (10.1017/erm.2022.3) 35098910 PMC9884763

[43] Santaballa A, Márquez-Vega C, Rodríguez-Lescure Á, et al. Multidisciplinary consensus on the criteria for fertility preservation in cancer patients. Clin Transl Oncol 2022 24 227–243. (10.1007/s12094-021-02699-2) 34635959 PMC8794945

[44] Himpe J, Lammerant S, Van den Bergh L, et al. The impact of systemic oncological treatments on the fertility of adolescents and young adults-a systematic review. Life 2023 13 1209. (10.3390/life13051209) 37240854 PMC10223569

[45] Stefansdottir A, Johnston ZC, Powles-Glover N, et al. Etoposide damages female germ cells in the developing ovary. BMC Cancer 2016 16 482. (10.1186/s12885-016-2505-9) 27510889 PMC4980800

[46] Walter JR, Lohman ME, Kundu SD, et al. A new fertility risk rating system for surgical, radiotherapy, and chemotherapy interventions used in testicular cancer. Transl Cancer Res 2016 5 S778–S781. (10.21037/tcr.2016.10.90)

[47] Gharwan H, Lai C, Grant C, et al. Female fertility following dose-adjusted EPOCH-R chemotherapy in primary mediastinal B-cell lymphomas. Leuk Lymphoma 2016 57 1616–1624. (10.3109/10428194.2015.1118476) 27183887 PMC7831158

[48] Levi M, Ben-Aharon I & Shalgi R. Irinotecan (CPT-11) treatment induces mild gonadotoxicity. Front Reprod Health 2022 4 812053. (10.3389/frph.2022.812053) 36303648 PMC9580821

[49] Osborne-Grinter M, Bianca OC, Sanghera J, et al. Fertility preservation techniques in neuro-oncology patients: protocol for a systematic review. JMIR Res Protoc 2023 12 e44825. (10.2196/44825) 37155238 PMC10203926

[50] Huyghe E, Zairi A, Nohra J, et al. Gonadal impact of target of rapamycin inhibitors (sirolimus and everolimus) in male patients: an overview. Transpl Int 2007 20 305–311. (10.1111/j.1432-2277.2006.00423.x) 17326771

[51] Bernard V, Bouilly J, Kramer P, et al. The tyrosine kinase inhibitor sunitinib affects ovulation but not ovarian reserve in mouse: a preclinical study. PLoS One 2016 11 e0152872. (10.1371/journal.pone.0152872) 27035144 PMC4818017

[52] Marino M, Cannarella R, Condorelli RA, et al. New insights of target therapy: effects of tyrosine kinase inhibitors on Male gonadal function: a systematic review. Clin Genitourin Cancer 2024 22 102131. (10.1016/j.clgc.2024.102131) 38901138

[53] Srigadha VK, Prabhash K, Noronha V, et al. Cabozantinib: a narrative drug review. Cancer Res Stat Treat 2023 6 74–87. (10.4103/crst.crst_9_23)

[54] Bastin J, Werbrouck E, Verbiest A, et al. Prospective evaluation of hypogonadism in male metastatic renal cell carcinoma patients treated with targeted therapies. Acta Clin Belg 2019 74 169–179. (10.1080/17843286.2018.1476115) 29774795

[55] Cathcart-Rake E, O’Connor JM, Jacobson A, et al. How (and why) to ask the older cancer patient about sexual health and sexual minority status. J Geriatr Oncol 2020 11 576–578. (10.1016/j.jgo.2019.08.003) 31447290 PMC7035152

[56] Sadovsky R, Basson R, Krychman M, et al. Cancer and sexual problems. J Sex Med 2010 7 349–373. (10.1111/j.1743-6109.2009.01620.x) 20092444

[57] Laumann EO, Paik A & Rosen RC. Sexual dysfunction in the United States. JAMA 1999 281 537. (10.1001/jama.281.6.537) 10022110

[58] Harrington CB, Hansen JA, Moskowitz M, et al. It’s not over when it’s over: long-term symptoms in cancer survivors – a systematic review. Int J Psychiatr Med 2010 40 163–181. (10.2190/PM.40.2.c) 20848873

[59] Zaid T, Burzawa J, Basen-Engquist K, et al. Use of social media to conduct a cross-sectional epidemiologic and quality of life survey of patients with neuroendocrine carcinoma of the cervix: a feasibility study. Gynecol Oncol 2014 132 149–153. (10.1016/j.ygyno.2013.10.015) 24145111 PMC4265467

[60] der Horst-Schrivers Ana van, van Ieperen E, Wymenga ANM, et al. Sexual function in patients with metastatic midgut carcinoid tumours. Neuroendocrinology 2009 89 231–236. (10.1159/000178754) 19033719

[61] Karppinen N, Lindén R, Sintonen H, et al. Health-related quality of life in patients with small intestine neuroendocrine tumors. Neuroendocrinology 2018 107 366–374. (10.1159/000494293) 30293074

[62] Defeudis G, Fioriti E, Palermo A, et al. A case of pheochromocytoma with negative MIBG scintigraphy, PET-CT and genetic tests (VHL included) and a rare case of post-operative erectile dysfunction. Hormones 2018 17 279–284. (10.1007/s42000-018-0037-1) 29860716

[63] Talvande B, Dorange A, Lecouflet M, et al. Tumeur carcinoïde ovarienne responsable d’une érythrose faciale permanente et de flushs au décours des coïts. Rev Med Interne 2016 37 775–778. (10.1016/j.revmed.2016.07.009) 27623329

[64] Yafi FA, Huynh LM, Ahlering T, et al. What is a “validated questionnaire”? A critical review of erectile function assessment. J Sex Med 2020 17 849–860. (10.1016/j.jsxm.2020.02.005) 32146130

[65] Davis SR. Sexual dysfunction in women. N Engl J Med 2024 391 736–745. (10.1056/NEJMcp2313307) 39167808

[66] Hatzichristou D, Rosen RC, Derogatis LR, et al. Recommendations for the clinical evaluation of men and women with sexual dysfunction. J Sex Med 2010 7 337–348. (10.1111/j.1743-6109.2009.01619.x) 20092443

